# Research on Mechanical Equipment Fault Diagnosis Method Based on Deep Learning and Information Fusion

**DOI:** 10.3390/s23156999

**Published:** 2023-08-07

**Authors:** Dongnian Jiang, Zhixuan Wang

**Affiliations:** 1 College of Electrical and Information Engineering, Lanzhou University of Technology, Lanzhou 730050, China; wzx_sll@126.com; 2 Key Laboratory of Gansu Advanced Control for Industrial Processes, Lanzhou University of Technology, Lanzhou 730050, China; 3 National Demonstration Center for Experimental Electrical and Control Engineering Education, Lanzhou University of Technology, Lanzhou 730050, China

**Keywords:** multi-sensor information fusion, fault diagnosis, theory of DS evidence fusion, 1DCNN

## Abstract

Solving the problem of the transmission of mechanical equipment is complicated, and the interconnection between equipment components in a complex industrial environment can easily lead to faults. A multi-scale-sensor information fusion method is proposed, overcoming the shortcomings of fault diagnosis methods based on the analysis of one signal, in terms of diagnosis accuracy and efficiency. First, different sizes of convolution kernels are applied to extract multi-scale features from original signals using a multi-scale one-dimensional convolutional neural network (1DCNN); this not only improves the learning ability of the features but also enables the fine characterization of the features. Then, using Dempster–Shafer (DS) evidence theory, improved by multi-sensor information fusion strategy, the feature signals extracted by the multi-scale 1DCNN are fused to realize the fault detection and location. Finally, the experimental results of fault detection on a flash furnace show that the accuracy of the proposed method is more than 99.65% and has better fault diagnosis, which proves the feasibility and effectiveness of the proposed method.

## 1. Introduction

As science and technology advance, intelligent systems are becoming increasingly dependent on multi-sensor information fusion technology; in the field of industrial machinery and equipment fault diagnosis, it plays a steadily more important role. In most complex industrial environments, a single sensor is used to acquire a specific piece of functional information from the mechanical equipment. However, the information obtained from a single sensor is limited by its own accuracy and performance and often cannot accurately describe the characteristics of the target fault, resulting in the observation signal being insufficient to reflect the operating condition of the equipment. Information fusion with multiple sensors increases the information transmission between sensors, improves the stability and accuracy of the system, and overcomes the influence of uncertain factors, such as interference. These are the key points in researching fault diagnosis in complex industrial equipment. The intelligent diagnosis and health maintenance of industrial equipment can increase equipment efficiency and reduce the operating and maintenance costs of enterprises, which have an important effect on carrying out real-time systematic and intelligent monitoring of industrial equipment.

A multi-source information fusion system for industrial fault diagnosis [1,2] can obtain valuable information which cannot be obtained from a single source of information. This is achieved through monitoring sensor information from multiple sources, comprehensively analyzing and processing it according to certain criteria, and completing diagnosis objectives. In recent years, research in the field of multi-source information fusion has focused on the complementarity of information from multiple sources. Fusion methods of feature-level and decision-level are applied to obtain valuable information for fault decisions and to obtain the most complete description of the objects observed by different sensors, which makes accurate fault analysis more effective [3]. Therefore, this paper proposes that mechanical devices can be fault-diagnosed using a multi-sensor fusion method, which combines multi-scale CNN and improved DS theory of evidence. Through the development of a multi-scale convolutional network, features of different fineness are extracted, and then the DS evidence theory integrates features from different scales, which enables the effective identification of faults and simplifies the diagnostic process. Through experimental comparison and analysis, by the superiority of this method, the efficiency and accuracy of fault diagnosis are improved.

As an intelligent and efficient fault diagnosis method, in the field of fault diagnosis, the fusion of information from multiple sources is applied more and more extensively. Its level of application is constantly improving. In the fault diagnosis field, it has become an important development direction. The rapid development of digital signal processing, production equipment, and systems are becoming increasingly complex. When mechanical equipment fails, it becomes increasingly difficult to analyze the reason for the fault and its location. In some cases, a single intelligent fault diagnosis technology cannot accurately assess the cause of the fault and may even result in the fault not being detected or not being detected correctly. If the operating condition of the system cannot be properly estimated, it will be difficult to make accurate decisions [4]. However, the perception performance is significantly increased by a multi-sensor system which has reliable diagnosis results [5].

In recent years, researchers have proposed many effective methods for processing the fault signals of mechanical device components. Lee proposed a joint transmission and detection scheme for IoT devices based on deep learning, which improved the detection capability of devices through joint detection using a multi-sensor fusion method [6]. Saxena proposed an analysis of the characteristics of fault data by continuous wavelet transform and performed the visual classification and identification of faults [7]. An integrated method comprising fuzzy entropy at multiple scales, selection of mode, and decomposition of empirical modes was proposed by Zhao for the extraction of fault features and realizing the diagnosis of faults in motor bearings [8]. An improved method for the decomposition of empirical modes for feature extraction, based on time-varying filtering, was proposed. In noise, it improves the robustness of analysis results and the ability to extract fault features [9]. Jiang proposed an adaptive rotating machine system for diagnosing faults under the influence of noise, which reduced the influence of noise and effectively extracted fault features [10]. However, the results of these methods of analysis are usually limited by prior knowledge, which is empirical to achieve a better extraction of fault features and better identification of fault types.

Artificial Intelligence (AI) technology is continuously developing, and research in the field of anomaly detection and decision making in sensor systems is gradually developing in this way also, enabling automatic error detection. An effective fault diagnosis method for a variable frequency-driven asynchronous motor based on machine learning was proposed by Gawde [11], which realized the fault diagnosis of the motor running state. Ali proposed a fault classification framework method for rotating machinery gears based on automatic data fusion, which solves the previous problem based on manual classification, realizes automatic fault classification through a machine learning algorithm, and improves robustness [12]. A rotating machine fault detection method using deep learning was proposed in [13], and this achieved a high diagnosis rate with fewer original training datasets. A method for fusing data from multiple sources for network attack and intrusion detection was proposed in [14] to obtain a larger amount of unique data to improve anomaly detection and system decision-making capabilities. In addition, there are other detection methods, including support vector machine [15], artificial neural networks [16,17], and random forest [18], which can also detect abnormal conditions and make appropriate decisions. Because of the various uncertainties influencing complex industrial environments, anomaly detection and decision making in multi-sensor systems usually depend on the accuracy of the data acquired. Once the measurement quality of the sensors decreases or faults occur, the overall diagnostic performance of the multi-sensor system is severely affected.

At present, combining multi-source information fusion technology with deep learning has become increasingly popular for fault diagnosis. Arellano-Espitia proposed a diagnosis method on the basis of multiple information source extraction and fusion in electromechanical systems, which can adaptively learn complex relationships in signals to characterize different fault states [19]. Huang proposed an information fusion method combining uncertain evidence and reinforcement learning, which improves the accuracy of fusion and solves the decision problem with low information, ignoring the decision implementation under the condition of a large amount of information [20]. Among the methods of real-time fault diagnosis and surveillance, a DS evidence theory combined with the principal component analysis fusion method was proposed by Yao for diagnosing rolling bearing faults and solving the low accuracy problem of fault classification [21]. Teng used 1DCNN to train signals from each sensing point and then improved the structural accuracy of damage detection by decision-level fusion [22]. Data fusion using improved DS evidence theory, which solved the incompleteness of measurement data from a single sensor and realized the information fusion of multiple measurement devices, was described in [23,24]. A diagnostic method for faults using a CNN, combined with sensor fusion, was proposed in [25], and it avoids manual feature extraction. A diagnostic method for faults using a one-dimensional long- and short-term convolutional network, combined with multi-sensor vibration signals, was proposed by [26,27]; this extracts the spatial–temporal characteristics of multi-sensor measurement signals and provides better fault diagnosis. Chen proposed the combination of Sparse Autoencoder Neural Network and Deep Belief Network for fault feature extraction and to identify the operating condition of the plant well [28]. In early fault diagnosis and recognition, Kiranyaz proposed adaptive one-dimensional CNN [29]. Li and Wang [30,31] proposed the combination of a multi-sensor and a CNN for fault diagnosis. In addition, fault diagnosis based on deep learning under different working conditions can effectively improve diagnostic accuracy. Such a deep CNN was used to diagnose bearing faults in a noisy environment and under different working loads in [32]. CNN is also used for data-driven fault diagnosis [33], etc. Analysis has shown that, although the deep learning method combined with the fusion of information from multiple sources can provide better diagnostic accuracy, the theory and system for fusing information from multiple sensors are not yet mature. There are still many problems with monitoring and diagnosing faults in applications, such as limited scenarios of sensor usage, low efficiency of multi-dimensional feature optimization and dimensionality reduction, and low model accuracy or generalization ability.

With the development of modern industry, there are many kinds of equipment and complex gears. The performance changes caused by faults in different devices, components, and locations are different, so it is necessary for multiple sensors to detect different data for decision making. Because of the incompatibility of data, traditional methods cannot be combined at data level and classifiers are not suitable for all data types. Therefore, data must be classified individually, combined with the classification results, to obtain accurate decision results. In view of this, a CNN combined with the DS evidence theory, this paper researches the fault diagnosis method with operable multi-sensor fusion. By using 1DCNN to classify data from different sensors in parallel and combining DS combination rules for optimization, fault information is obtained from mechanical devices. The main contributions of the proposed method include the following: methods of fault diagnosis based on 1DCNN network are adopted, and the multi-scale parallel processing of different sensor data is used to solve the task requirements under different working conditions. Compared with other methods, there is faster detection efficiency. Moreover, improved DS evidence theory is combined with the 1DCNN network so that the detection efficiency in fault isolation is higher and the accuracy of fault classification is improved.

The rest of the paper is organized as follows: Section 2 describes the theoretical knowledge on convolutional neural networks. Section 3 depicts the multi-sensor information fusion’s fault diagnosis method based on the DS evidence theory. Section 4 is about experimental verification and analysis, which is an overview of the experimental details. Finally, Section 5 is the conclusion.

## 2. Convolutional Neural Network

**Figure 1:**
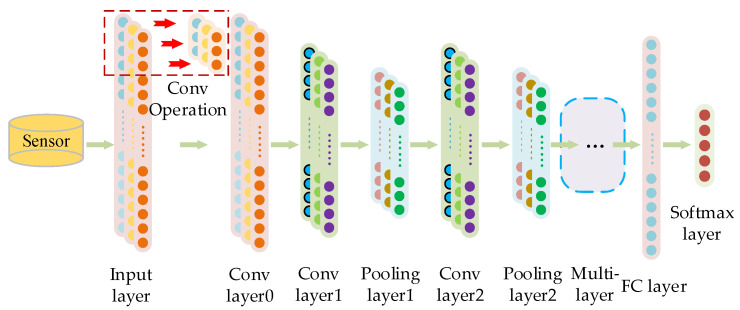
Structure diagram of 1DCNN.

A CNN is an important part of a deep neural network, which consists of trainable multilevel architecture and is widely used for its good feature extraction ability. A CNN generally consists of five modules: input layer, convolutional layer, pooling layer, fully connected layer, and output layer. The 1DCNN can process and retain the original data characteristics. Each stage of the 1DCNN contains a pooling layer and a convolutional layer. As shown in Figure 1, the fault features are extracted by multi-stage alternating operations, and faults are classified by the fully connected layer and classifier.

### 2.1. One-Dimensional Convolutional Neural Network

The input layer of the 1DCNN processes the raw multidimensional data in a standardized form and standardizes the input data before importing the raw data into the 1DCNN. An algorithm’s operational efficiency and learning performance can be improved by standardizing the input features.

The convolutional layer of the 1DCNN realizes local connections and weight sharing through the convolution kernel. The convolution kernel along the horizontal and longitudinal sliding of the input time series in the convolution layer. One-dimensional data, such as vibration, acoustics, and temperature, can be processed then the size of the convolution kernel is set to 1×k, which must be within the limit of the length of the input samples. The output features are constructed using nonlinear activation functions. Multiple input features are convoluted into each layer’s output. The process of extracting features through a convolution kernel is described as follows:

(1) $$\begin{array}{l} x_{i}^{l+1}=\sum_{i\in M_{j}}w_{ij}^{l}\times x_{i}^{l}+b_{j}^{l}\\ y_{i}^{l+1}=f(x_{i}^{l+1}) \end{array}$$

where $x_{i}^{l+1}$ represents the *i*-th output of layer *l*; (*) represents a convolutional operation; $M_{j}$ represents the *j*-th convolutional region in the *l* layer; $x_{i}^{l}$ represents the *i*-th feature; $b_{j}^{l}$ represents the *j*-th offset of *l* layer; $w_{ij}^{l}$ represents, in the *l* layer, the *j*-th weight value of the *i*-th convolutional kernel; and *f* is the activation function.

After the convolutional operation, the corresponding output features can be obtained by the nonlinear transformation processing of the operation result by activating the function. The choice of network activation function affects the training time, especially the performance of large datasets. The specific expression is

(2) f(x)=max(0,x)

where *f*(*x*) denotes the value of the output obtained after activating the function; *x* denotes the value of the input.

Using a pooling layer reduces the feature’s data dimension, behind the convolutional layer, and preserves the important feature information whilst reducing the feature dimension. This paper uses maximum pooling, which can be expressed as follows:

(3) $$y_{j}^{l+1}=\underset{k\in M}{\max}(y_{j}^{l})$$

where *M* represents the pooled area of neurons; $y_{i}^{l}$ denotes, in the *l* layer, the value of the *j*-th feature; and $y_{i}^{l+1}$ represents the maximum value after pooling.

Behind the pooling layer is the fully connected layer, which integrates the extracted local features. The output can be expressed as follows:

(4) $$z_{j}^{l}=f(\sum_{i=1}^{M}x_{i}^{l-1}w_{j,i}^{l}+b_{j}^{l})$$

where *f* is the activation function; *w* represents the weight value; $x_{i}^{l-1}$ represents the length *M* of a one-dimensional input; *j* represents that there are *N* neurons in the fully connected layer; $z_{j}^{l}$ represents the output of each neuron; and $w_{j,i}^{l}$ denotes bias.

The Softmax classifier is used as the output layer and uses the category or probability form as the recognition result of the output model. Softmax converts the extracted features into a probability distribution and uses the value of the probability distribution to estimate the possibility of sample $x_{i}$ belonging to category $y_{i}$. The Softmax classification process can be expressed as follows:

(5) $${\tilde{y}}_{j}=\mathrm{soft}\max(z_{j}^{l})=\frac{\exp(z_{j}^{l})}{\Sigma_{k=1}^{C}\exp(z_{j}^{l})}$$

where $z_{j}^{l}$ represents the node value of the *j*-th neuron; *C* represents the total number of categories; and ${\tilde{y}}_{j}$ represents the value of classification.

Batch normalization (BN) uses the computation of the mean and variance estimate on small training sets to adjust the scale of the input features, improving the generalization ability of the network, speeding up the training process of the model, and reducing the transfer of internal covariates. The specific steps of the BN layer are shown in Equations (6)–(9):

(6) $$\mu_{B}=\frac{1}{n}\sum_{i=1}^{n}x_{i}$$

(7) $$\sigma_{B}^{2}=\frac{1}{n}\sum_{i=1}^{n}{\left(x_{i}-\mu_{B}\right)}^{2}$$

(8) $$x_{i}^{’}=\frac{x_{i}-\mu_{B}}{\sqrt{\sigma_{B}^{2}+\varepsilon }}$$

(9) $$y_{i}=\gamma_{i}\times x_{i}^{’}+\beta_{i}$$

In input data distribution, γ represents the variance and β represents the offset.

The output results are obtained by forward propagation during the training process of 1DCNN. The model output and sample labels are used to construct a cross entropy loss function. The back propagation algorithm is used for layer-by-layer feedback, and each network layer is updated using the gradient descent algorithm. By repeating the two steps of forward and backward propagation, the weighting parameters and the optimization loss function are continuously updated until the recognition result reaches a satisfactory recognition rate or the iteration number reaches a maximum.

### 2.2. Multi-Scale One-Dimensional Convolutional Neural Network

The 1DCNN can identify the faults of industrial machinery and equipment by extracting fault features using a convolution kernel of a single size. However, when only a one-dimensional convolutional kernel is used in a single-layer convolutional network, the local subtleties may not be considered, resulting in low model accuracy and poor generalization performance; the extracted information is incomplete, and the learning effect is affected. The multi-scale 1DCNN possesses stronger feature learning ability. It can extract different degrees of features from complex signals and perform fine characterization of signal features, so that the feature expression is more adequate, which enables more accurate identification and classification of faults in mechanical devices.

**Figure 2:**
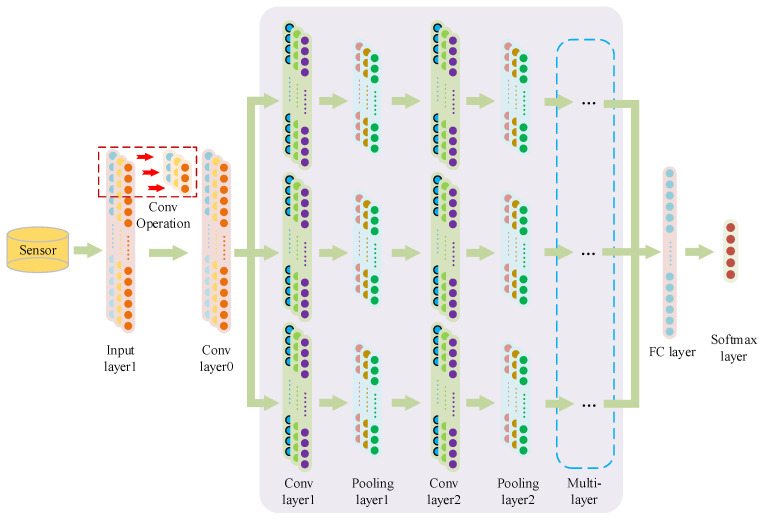
Structure diagram of multi-scale 1DCNN.

Therefore, based on the basic principle of a CNN, a multi-scale 1DCNN is proposed in this paper. The structure of the multi-scale 1DCNN is shown in Figure 2. Using the original time-domain signal as input, data abstracted from their original source can be effectively learned, while the original characteristic signal detected by the sensor is preserved. The sensor signal is preprocessed by the input layer and then input into the multi-scale 1DCNN. To obtain larger features, the first layer uses a large convolution kernel in the model. After the first convolutional layer, three scale branches are set and different sizes of three convolution kernels are used to convolute the signal in parallel. Each branch includes a multi-level pooling layer and convolutional layer to achieve signal feature extraction with different scales of fineness, thus improving the accuracy of the diagnostic. Finally, the features extracted from the three scales were flattened, then input into the fully connected layer for classification.

## 3. Multi-Sensor Information Fusion Based on Dempster–Shafer Evidence Theory

### 3.1. Dempster–Shafer Evidence Theory

The DS evidence theory is a kind of imprecise reasoning theory, which should be the first used in expert systems to deal with uncertain information. Dempster first proposed this theory in the field of statistical reasoning, which was later improved by Shafer as the main framework for modeling cognitive uncertainty theory. This theory allows for the combination of evidence from different sources [34,35]. In the absence of prior information, the uncertain information can be fused to arrive at a decision outcome. Researchers have conducted a large number of studies on DS, solving the paradoxical problem of synthesizing multiple conflict pieces of evidence during evidence theory synthesis [36,37,38,39,40].

The DS evidence theory gives an initial value for assigning the degree of confidence for each body of evidence, i.e., basic probability assignment (BPA), and finds the degree of support through mathematical operations. The basic probability distribution function is denoted as *m*. It completes the mapping from $2^{\Theta }$ to [0, 1] for any subset A in frame $\Theta =(\theta_{1},\theta_{2},\cdots ,\theta_{n})$ and satisfies Equation (10):

(10) $$\left\{ \begin{array}{l} m(\emptyset )=0\\ \sum_{A\subset \Theta }m(A)=1\\ m(A)\in [0,1] \end{array}\right.$$

In the identification framework, the degree of confidence of the empty set is 0, and the sum of the degrees of confidence of all subsets *A* is 1. *A* may contain only one element or it may contain many elements, and *m*(*A*) is determined by these elements (they are the body of evidence *E* of subset *A*).

In the DS theory, plausibility function (*Pl*) and belief function (*Bel*) are the two main functions. For any proposition, there is doubt, as well as true and false.

The plausibility function is used to describe the degree that the proposition is not necessarily false, i.e., the maximum possibility that it can be true. On Θ, the plausibility function $Pl:2^{\Theta }\to [0,1]$ describes the proposition where *A* satisfies $\forall A\in 2^{\Theta }$ and the sum of the mass functions of the intersection subset of proposition *A* and proposition $C\in 2^{\Theta }$. *Pl* is described by (11):

(11) $$Pl(A)=\sum_{C\cap A\neq \emptyset }m(C)$$

The belief function is used to describe the degree to which the proposition must be true. On Θ, the belief function $Bel(A):2^{\Theta }\to [0,1]$ describes the proposition where *A* satisfies $\forall A\in 2^{\Theta }$ and the sum of the mass functions of all subsets in the proposition *A*. *Bel* is described by (12):

(12) $$Bel(A)=\sum_{C\subseteq A}m(C)$$

*Bel*(*A*) is the lower bounds of the uncertainty of proposition *A*; *Pl*(*A*) is the upper bounds of the uncertainty of proposition *A* (namely *Bel*(*A*) < *P*(*A*) < *Pl*(*A*)).

The DS synthesis rule performs orthogonal operations on the evidence obtained from different data sources to maintain confidence in the proposition through the accurate fusion of the bodies of evidence.

For the traditional DS evidence theory, when there is evidence from *n* different sources, the DS synthesis rule is shown by (13), and the normalization constant *K* is shown in (14):

(13) $$(m_{1}⊕m_{2}⊕\cdots ⊕m_{n})(A)=\frac{1}{K}\sum_{A_{1}\cap \cdots \cap A_{n}=A}m_{1}(A_{1})m_{2}(A_{2})\cdots m_{n}(A_{n})\;$$

(14) $$\begin{array}{ll} K & =1-k=\sum_{A_{1}\cap \cdots \cap A_{n}\neq \emptyset }m_{1}(A_{1})m_{2}(A_{2})\cdots m_{n}(A_{n})\\ & =1-\sum_{A_{1}\cap \cdots \cap A_{n}=\emptyset }m_{1}(A_{1})m_{2}(A_{2})\cdots m_{n}(A_{n}) \end{array}$$

Here, k=1−K is defined as a conflict factor whose range of values [0, 1] represents the degree of conflict between different evidence. When *k* is relatively large, the conflict between the different evidence is more serious. When *k* is lesser, there is a good consistency between the evidence.

### 3.2. Improved Dempster–Shafer Evidence Theory

In the traditional DS theory, when multiple bodies of evidence are synthesized, contradictory evidence leads to the phenomenon that the actual result of the synthesis contradicts intuition. With the aim of correcting the shortcomings of the traditional DS theory of evidence, domestic and foreign experts have conducted a large amount of research into improvement methods in recent decades. There are three main categories: the modification of the combination rules, the modification of the model, and the modification of both the model and the composition rules. Since the combination rules of the traditional DS evidence theory have a clear mathematical meaning, calculating the mutual support between the cosine similar bodies of evidence was chosen to modify the model in this paper. The cosine similarity is accumulated and normalized to obtain evidence credibility. Credibility is a weighted coefficient of the weighted average, which replaces the original evidence. Finally, the DS rules for combining evidence were used for synthesis. Using the method of weighted average to process evidence, not only are the shortcomings of the traditional DS theory of evidence effectively eliminated but the credibility to distribute the weight of the body of evidence is also fully used, so that the improved model is more reasonable and the improvement effect is more ideal.

It is assumed that there are *n* independent evidence bodies and *k* unrecognized states $\left\{X_{1},X_{2},\cdots ,X_{k}\right\}$ which are monad sets. Vector ${\vec{m}}_{i}$ is used to represent the *i*-th evidence body, and $m_{iw}$ is used to represent the BPA of the *w*-th unrecognized state in the evidence body ${\vec{m}}_{i}$. The improvement method steps are as follows:

Step 1: Find the cosine similarity between the evidence bodies.

The cosine similarity between any two bodies of evidence ${\vec{m}}_{i}$ and ${\vec{m}}_{j}$ is

(15) $$Sim({\vec{m}}_{i},{\vec{m}}_{j})=\frac{{\vec{m}}_{i}\cdot {\vec{m}}_{j}}{\left\|{\vec{m}}_{i}\right\|\times \left\|{\vec{m}}_{j}\right\|}=\frac{\sum_{w=1}^{k}m_{iw}m_{jw}}{\sqrt{\sum_{w=1}^{k}m_{iw}^{2}}\sqrt{\sum_{w=1}^{k}m_{jw}^{2}}}$$

The similarity matrix is obtained by traversing the three evidence bodies and calculating the similarity between any two evidence bodies:

(16) $$\left[\begin{array}{ccc} 1 & S_{12} & S_{13}\\ S_{21} & 1 & S_{23}\\ S_{31} & S_{32} & 1 \end{array}\right]$$

Step 2: Find the mutual support between each evidence body.

The support degree analyzes the support degree of other evidence bodies to ${\vec{m}}_{i}$ except ${\vec{m}}_{i}$ itself:

(17) $$Sup({\vec{m}}_{i})=\sum_{\begin{array}{l} j=1\\ j\neq i \end{array}}^{n}Sim({\vec{m}}_{i},{\vec{m}}_{j})$$

Step 3: Find the weight coefficient.

The reliability ($Crd({\vec{m}}_{i})$) is obtained by normalization $Sup({\vec{m}}_{i})$. $Crd({\vec{m}}_{i})$ is the weight coefficient and the calculation is

(18) $$Crd({\vec{m}}_{i})=\frac{Sup({\vec{m}}_{i})}{\sum_{i=1}^{n}Sup({\vec{m}}_{i})}=\frac{\sum_{\begin{array}{l} j=1\\ j\neq i \end{array}}^{n}Sim({\vec{m}}_{i},{\vec{m}}_{j})}{\sum_{i=1}^{n}\sum_{\begin{array}{l} j=1\\ j\neq i \end{array}}^{n}Sim({\vec{m}}_{i},{\vec{m}}_{j})}$$

Step 4: Find the weighted average evidence.

(19) $$m^{\prime }(X_{w})=\sum_{i=1}^{n}m_{iw}Crd({\vec{m}}_{i}),w=1,2,\cdots ,k$$

where $m^{\prime }$ is the weighted average evidence; $m^{\prime }(X_{w})$ is the BPA of the *w*-th unidentified state in the weighted average evidence $m^{\prime }$.

Step 5: The DS combination evidence rule was adopted to perform *n* − 1 self-combinations of *n* weighted average evidence $m^{\prime }$ and to obtain the final synthesis result:

(20) $$m^{\prime }(X)=m^{\prime }⊕m^{\prime }⊕\cdots ⊕m^{\prime }=\left\{ \begin{array}{l} \frac{\sum_{\cap X_{w}=X}\prod_{i=1}^{n}{m^{\prime }}_{i}(X_{w})}{1-\sum_{\cap X_{w}=\Phi }\prod_{i=1}^{n}{m^{\prime }}_{i}(X_{w})},X\neq \Phi \\ 0,A=\Phi \end{array}\right.$$

where ${m^{\prime }}_{i}(X_{w})$ represents BPA, which is *w*-th unidentified state in the *i*-weighted average evidence.

### 3.3. Multi-Sensor Information Fusion

**Figure 3:**
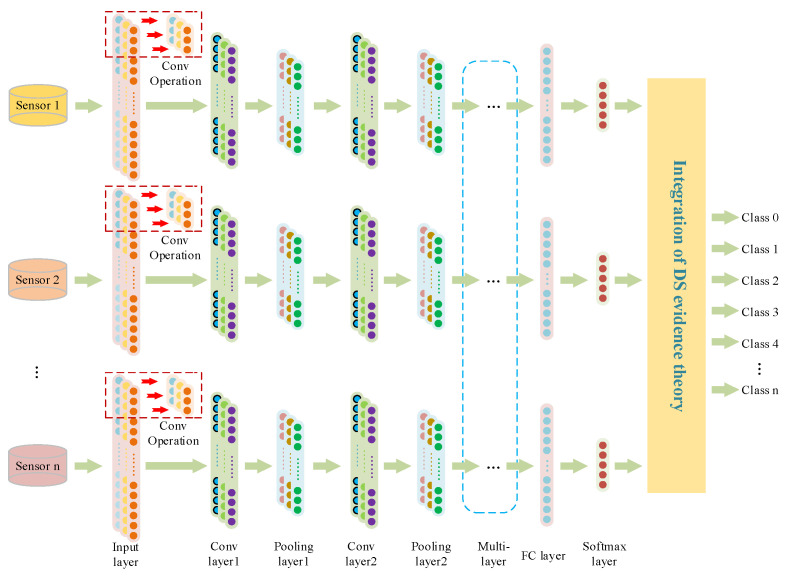
Structure diagram of multi-sensor fusion model.

Different sensors acquired information, which is integrated by the DS evidence theory for comprehensive analysis and to achieve more accurate statistical recognition. Compared to a single sensor, the accuracy and fault tolerance of a multi-sensor system is much better. In this paper, the structure of the multi-sensor fusion decision method is proposed. Firstly, the feature is extracted by the one-dimensional convolution module; the obtained feature data are then flattened and aggregated in the fully connected layer. Then, from the 1DCNN model obtained, the features are classified by Softmax and the DS synthesis rules are used to fuse the features; the diagnosis results are then obtained. Finally, the 1DCNN convolutional structure is replaced by the multi-scale 1DCNN structure to achieve the final diagnosis in the same way, as is shown in Figure 3.

**Figure 4:**
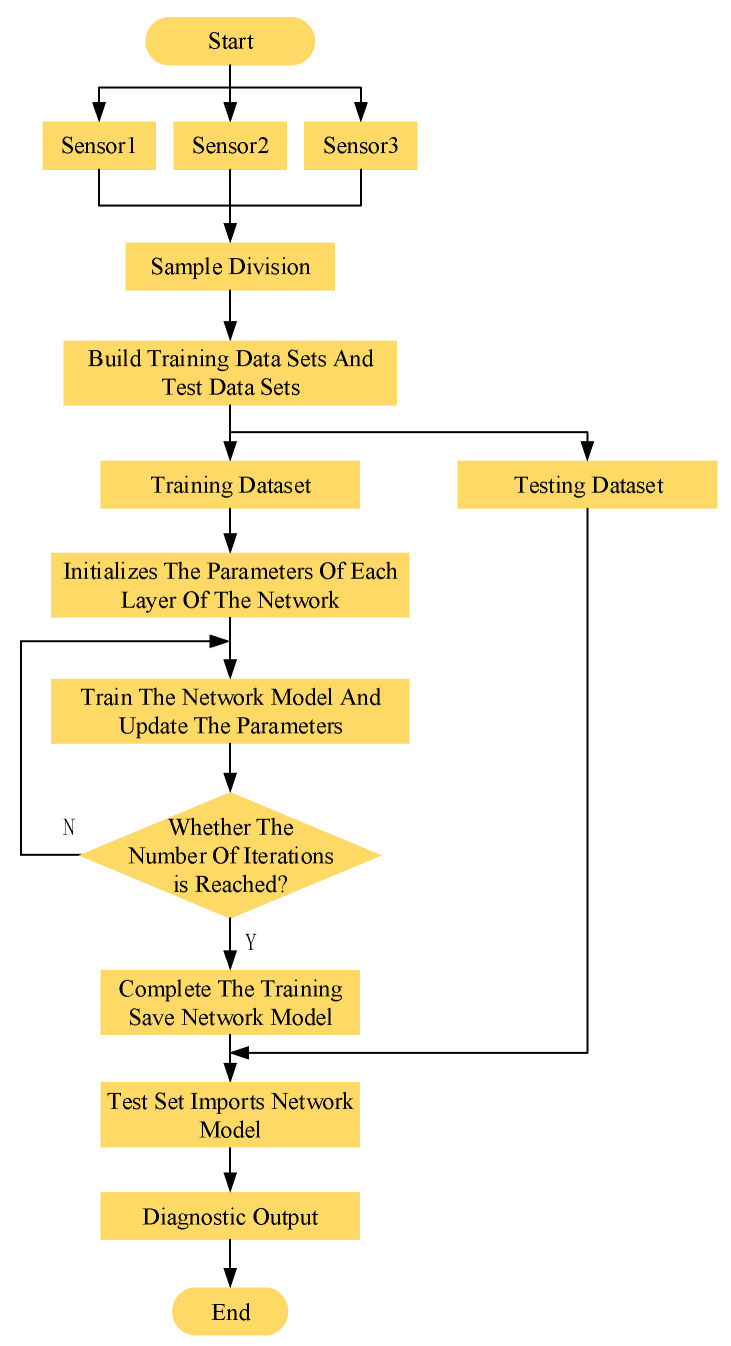
Flow chart of network model training.

The fault diagnosis of the mechanical equipment was performed by constructing a 1DCNN model. Figure 4 shows the training flow diagram of the network model. For multiple measurement points of the system, one-dimensional signals of the mechanical equipment at different faults were measured by a variety of sensors. These one-dimensional signals are classified according to different faults and training sets. The testing sets are divided by all labeled data, as input into the 1DCNN model. By initializing the network parameters and selecting the optimal batch size and learning rate, the training set is input for self-learning. The trained 1DCNN is then verified by the testing set for identifying and classifying device faults.

The input parameters of the multi-sensor fusion method include the initialization value of the network, the dataset, the anticipated loss rate of training termination, and the iteration times. Table 1 shows the pseudocode for the algorithm. The training subset and testing subset are divided by all labels and data. An epoch is the maximum iteration time. $w_{i}$ is weight in the *i*-th convolutional layer and pooling layer and is randomly initialized. $E_{r}$ is the expected error at the end of the training phase, obtained by empirical knowledge. *W* is the weight, and *b* is bias. The output parameters include the result of each iteration and confusion matrix. $y_{i}$ is the output result of *n* training iterations of the 1DCNN model at each scale. $M_{ij}$ is the confusion matrix for each 1DCNN at each scale. $J_{i}(\theta )$ is the error of each 1DCNN after each iteration. $J_{j}(\theta )$ is the error of DS after each iteration.

After setting the initialization parameters, the training starts in the while loop, and multiple sensor data are sent to the 1DCNN model in parallel with the training. Algorithm 1 illustrates the main structure of the multi-sensor fusion model. It is used to describe the parameters and weights of the model in the training and updating stages. After each iteration of model training, the model prediction (${\tilde{y}}_{i}$) and model loss ($J_{i}(\theta )=\frac{1}{2}\sum_{n=1}^{N}{\left({\tilde{y}}_{i}-y_{p}\right)}^{2}$) are calculated. Then, the confusion matrix of each scale 1DCNN is calculated and the output results are obtained. The combination rules of the DS theory are merged according to the Equations (15)–(20) to obtain the predicted value.

Finally, the DS loss of $J_{j}(\theta )=\frac{1}{2}\sum_{n=1}^{N}{\left({\tilde{y}}_{i}-y_{pd}\right)}^{2}$ is calculated using the predicted value, and the parameters of the gradient aggregation and the past gradient of the Adam optimizer are calculated using the 1DCNN and the DS loss. The specific process of the Adam algorithm is shown in (21):

(21) $$\begin{array}{l} \theta_{t}=\theta_{t-1}-\alpha {\hat{m}}_{t}/(\sqrt{{\hat{v}}_{t}}+\varepsilon )\\ {\hat{m}}_{t}=m_{t}/(1+\beta_{1}^{t})\\ m_{t}=\beta_{1}(m_{t-1})+(1-\beta_{1})(\rho J_{i}(\theta )+\rho J_{j}(\theta ))\\ {\hat{v}}_{t}=v_{t}/(1-\beta_{2}^{t})\\ v_{t}=\beta_{2}v_{t-1}+(1-\beta_{2}){\left(\rho J_{i}(\theta )+\rho J_{j}(\theta )\right)}^{2} \end{array}$$

where ρ is the weight of the error of 1DCNN and DS; $y_{pd}$ is the prediction of the model; $\beta_{1}$ and $\beta_{2}$ are exponential decay rates used to control the influence of the weight allocation and the gradient square, respectively; ε is a very small constant that has little effect on the algorithm and avoids division by 0; α is the learning rate that controls the update rate of the weights during backpropagation; $v_{t}$ represents the exponential square of the past gradient; $m_{t}$ represents the exponentially weighted average of the past gradient index; and ${\hat{m}}_{t}$ and ${\hat{v}}_{t}$ are the correction values of the corresponding terms. During the training process, the model parameter θ is updated through the above steps for each iteration of (21) until the network error converges.
**Algorithm 1:** Pseudocode of Multi-sensor fusion model.**Input:** Initialize iteration variable; $w_{i}$, W,b: These parameters are set randomly **Require:** The training sample set is formed by using sliding window: *y_test, y_train*, *X_test*, *X_train* 1: **while** *n*< Epochs and (ρ$J_{i}(\theta )$ + (1 − ρ)$J_{j}(\theta )$) < $E_{r}$ **do** 2: **for** each 1DCNN in Dataset **do** 3:  **for** all *X*_train **do** 4:    $y_{pd}$ is obtained from *X*_train by using Formulas (15)–(20) 5:  **end for** 6:  Calculate ${\tilde{y}}_{i}$ 7:  Calculate confusion matrix $M_{ij}$ 8:  Set up loss function J(θ) and Calculate $J_{i}(\theta )$ 9: **end for** 10: Calculate $J_{j}(\theta )$ 11: Using the Adam algorithm, find global optimal solution of gradient descent as fast as possible 12: $w_{i}$, W,b are updated using equation 13: *n* + = 1 14: **end while** **Output:** ${\tilde{y}}_{i}$


## 4. Experimental Verification and Analysis

### 4.1. One-Dimensional Convolutional Neural Network Diagnostic Analysis

#### 4.1.1. Establishment of Diagnostic Model

**Figure 5:**
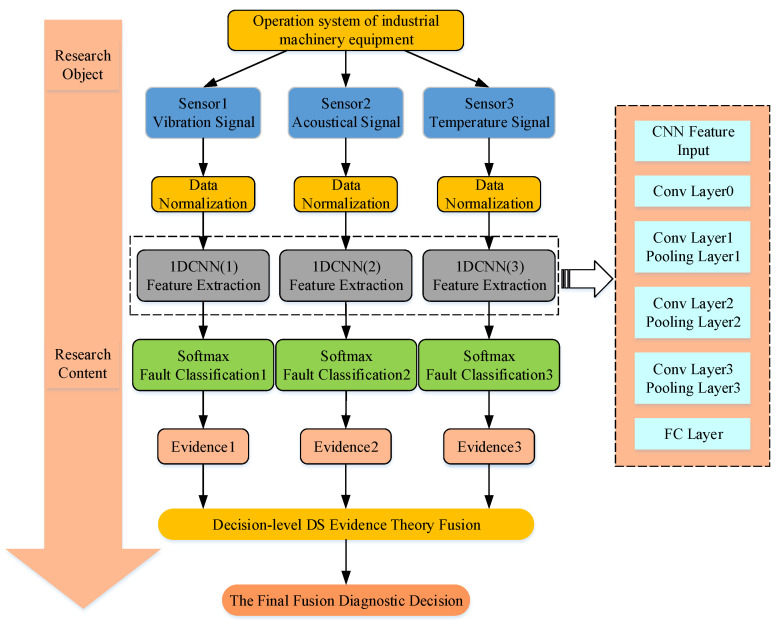
Architecture diagram of the system.

The architecture is shown in Figure 5. The diagnosis of faults in the blower of a flash furnace used in nickel smelting by the method proposed in this paper was undertaken. For the safe operation of a flash furnace, a low desulfurization rate should be avoided, and equipment damage and even casualties can be caused by abnormal fans; several sensors are used to monitor the fan’s operation and adjust the speed of the fan or stop the operation of the fan according to the data collected. There are many types of mechanical components in the flash furnace system, and there are limitations in fault diagnosis analysis based on only one signal source. The signals from multiple sources, such as vibration, acoustic, and temperature, generated in the event of a system fault complement each other, resulting in an improvement in the fault diagnosis rate. To demonstrate the method’s effectiveness, three types of sensors acquired during plant operation were selected from the diagnostic model for simulation verification.

#### 4.1.2. Experimental Data

In order to ensure the diversity of experimental data, five different working conditions were simulated in the experiment, using a vibration sensor to detect the vibration signal, an acoustic sensor to detect the acoustic signal, and a temperature sensor to detect the temperature signal. To obtain objective results, sampling was used to obtain experimental data from the original data. The total number of samples for each type of defect was 4000, which corresponded to 4000 vibration samples, 4000 acoustic samples, and 4000 temperature signals at the corresponding time, for a total of 20,000 vibration samples, 20,000 acoustic samples, and 20,000 temperature signals. According to the ratio of 4:1, a random selection of 75% of the dataset was used for training, and the remaining 25% of the dataset was used for testing. The data were fed into the model for learning and training. The sample data is shown in Table 2.

The common gearbox fault types are wear, shedding, tooth breakage, eccentric wear, and skew; common bearing fault types are wear, fatigue, shedding, sediment, and eccentricity; common types of generator fault are winding burnout, brush wear, bearing failure, mechanical component damage, and unstable motor operation.

For generator faults, bearing faults, and gearbox faults, the vibration signals of various faults were collected by vibration sensors for analysis. The experiment simulated one normal condition and five different working conditions with a sampling frequency of 100 Hz. In total, 4700 data points were collected under each working condition, and 28,200 data points, including each type of fault, were collected. The sample data for each type of fault is shown in Table 3.

#### 4.1.3. Model Training and Parameters

In this paper, the 1DCNN network model consists of three scale submodels, where each individual submodel includes a large convolutional layer, three groups of convolution and maximum pooling, and a flattening layer. Each submodel finally converges with the fully connected layer. In the end, Softmax outputs the classification result.

Every convolution module of the three scales adopts the same parameter setting. The parameters of the convolution and pooling layer in each branch are the same. All convolutional layers in the model use the activation function Relu, and the filling method is the same. The dropout parameter is set to 0.5. Softmax has five output neurons, corresponding to several fault states. Table 4 shows the specific structural parameters of the 1DCNN.

#### 4.1.4. Experimental Effect Analysis

**Figure 6:**
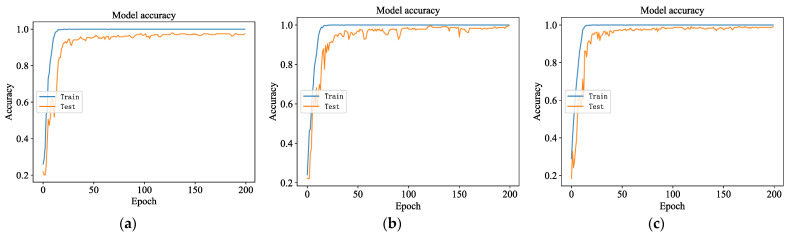
Accuracy in the training process. (**a**) Accuracy of vibration sensor; (**b**) accuracy of acoustic sensor; (**c**) accuracy of temperature sensor.

**Figure 7:**
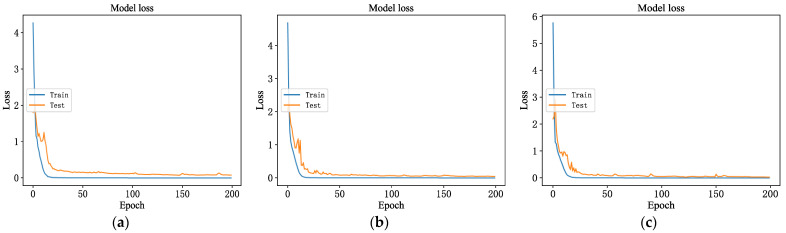
Loss value in the training process. (**a**) Loss value of vibration sensor; (**b**) loss value of acoustic sensor; (**c**) loss value of temperature sensor.

The accuracy and loss values of the 1DCNN network model in the training process are shown in Figure 6 and Figure 7. As the iteration times increase, the accuracy curve is on an upward trend, while the loss curves are on a downward trend; the model performance becomes better and better. After several iterations, in the training set, the model’s loss value finally approaches zero, and the recognition accuracy reaches 100%. In the test set, the model’s loss value gradually decreases and then remains in a state of fluctuation. The recognition accuracy increases rapidly, from about 20% at the beginning, and then stabilizes gradually. The highest recognition accuracy during the training process was 98%, with about 30 iterations.

**Figure 8:**
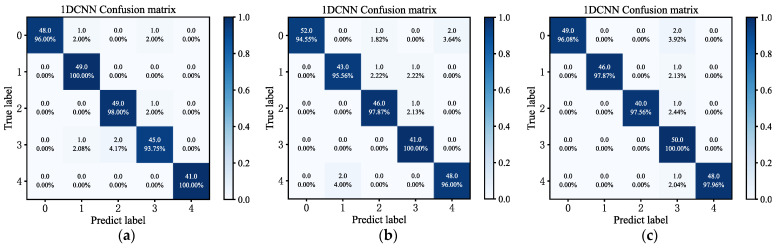
Confusion matrix for fault diagnosis. (**a**) Confusion matrix of vibration sensor; (**b**) confusion matrix of acoustic sensor; (**c**) confusion matrix of temperature sensor.

Figure 8 shows the confusion matrix. The values on the matrix grid represent the number and proportion of predicted correctness in each type of sample. Figure 8 shows that there are 41 and 49 correct predictions in the normal state 1 and state 4 categories in Figure 8a, which is 100%. Of the predictions in the state 0 category, 48 were correctly predicted, which corresponds to 96%; of the predictions in the state 2 category, 49 were correctly predicted, which corresponds to 98%. Of the predictions in the state 3 category, 45 were predicted correctly, corresponding to 93.75%. Figure 8b,c were analyzed using the same method. The model can effectively identify the five states of the flash furnace.

**Figure 9:**
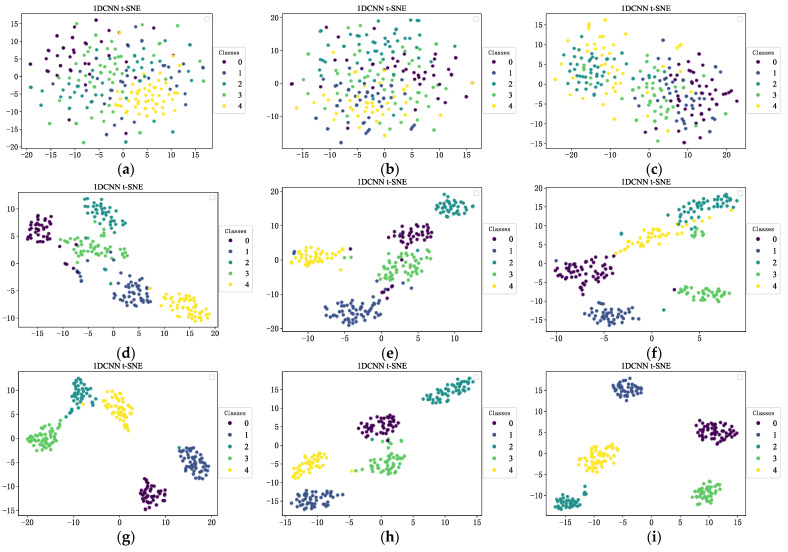

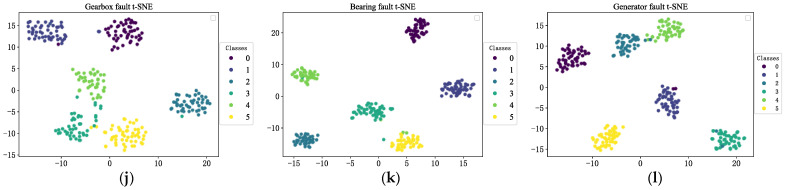
t-SNE dimensional reduction visualization. (**a**) t-SNE of original vibration signal; (**b**) t-SNE of original acoustic signal; (**c**) t-SNE of original temperature signal; (**d**) t-SNE of single scale vibration signal; (**e**) t-SNE of single scale acoustic signal; (**f**) t-SNE of single scale temperature signal; (**g**) t-SNE of multi-scale vibration signal; (**h**) t-SNE of multi-scale acoustic signal; (**i**) t-SNE of multi-scale temperature signal; (**j**) t-SNE of gearbox fault; (**k**) t-SNE of bearing fault; (**l**) t-SNE of generator fault.

To more intuitively study the classification effect of flash furnace states in each layer of the CNN, the T-distributed stochastic neighbor embedding (t-SNE) algorithm is used to visualize model output signals in two dimensions, as shown in Figure 9. Each feature is essentially separated and aggregated and has a good clustering effect. Finally, the five types of states can be well identified, with obvious linear boundaries.

In Figure 9j–l, each feature is essentially separated and aggregated and has a good clustering effect. Finally, the six types of states can be well identified, with obvious linear boundaries.

#### 4.1.5. Comparative Study of Models

**Figure 10:**
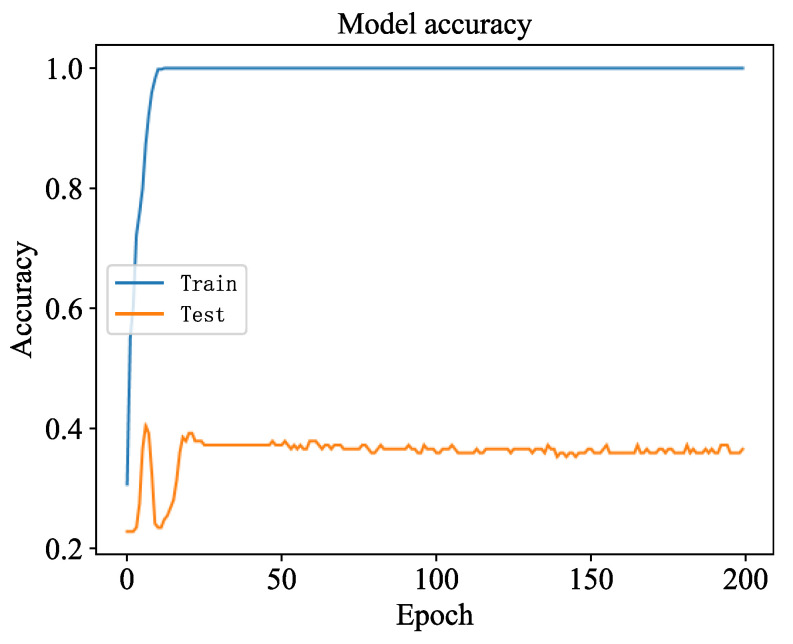
Accuracy of the LSTM model.

As is shown in Figure 10, data from one sensor were analyzed in a comparative experiment. In the training process, the accuracy and loss values of the long short-term memory network (LSTM) were calculated. The model training stopped in advance, after about 30 iterations. Compared with the diagnosis of the 1DCNN, the recognition speed of the LSTM state is slower, and the recognition effect is worse. From Figure 10, the recognition accuracy of the LSTM on the verification set remains unchanged at about 37% after about 30 iterations; the loss value is also large, and the decline rate is extremely slow, which proves that the fault diagnosis ability of the LSTM is poor.

**Figure 11:**
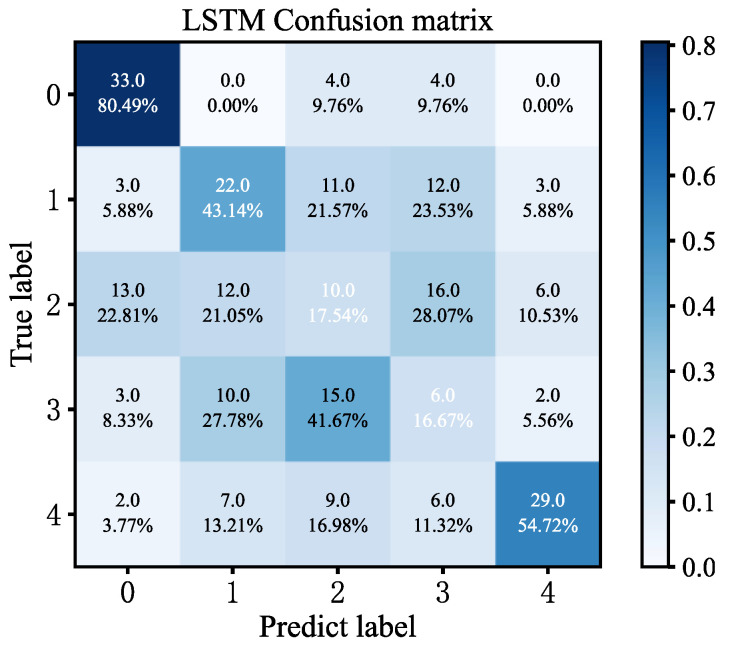
LSTM confusion matrix.

Figure 11 shows the confusion matrix. From Figure 11, there are 33 correct predictions in the category of state 0, which corresponds to 80.49%. Of the predictions in the state 1 category, 22 were correctly predicted, which corresponds to 43.14%. Of the predictions in the category of state 2, 10 were predicted correctly, which corresponds to 17.54%; of the predictions in the category of state 3, 6 were predicted correctly, which corresponds to 16.67%; of the predictions in the category of state 4, 29 were predicted correctly, which corresponds to 54.72%. From the test results, the LSTM model cannot effectively identify the five states of the flash furnace.

**Figure 12:**
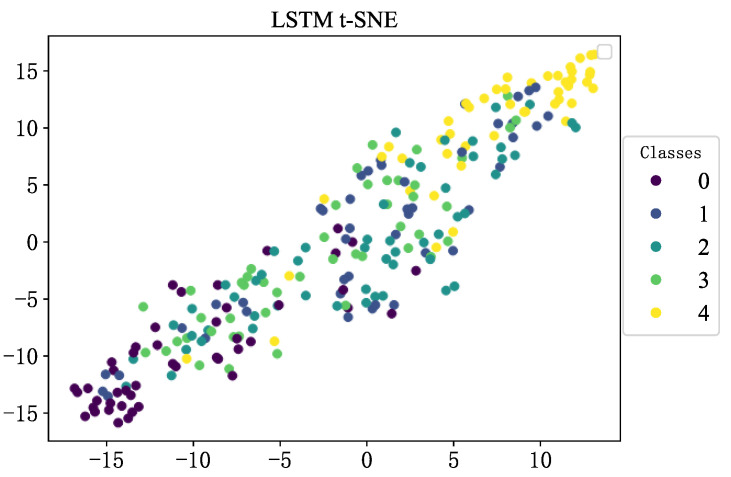
t-SNE visualization for LSTM.

To more intuitively study the classification effect of flash furnace states in the LSTM model, the T-distributed stochastic neighbor embedding (t-SNE) algorithm is used to visualize model output signals in two dimensions, as shown in Figure 12. The individual features are not completely separated and aggregated; the clustering effect is small. Finally, the five types of fault conditions are not separated, so the recognition effect of the model is low.

### 4.2. Dempster–Shafer Fusion Diagnosis Analysis

#### 4.2.1. Fault Diagnosis Evaluation Criteria

The evaluation criteria for fault diagnosis are expressed by a confusion matrix which, in supervision learning, is a visual tool. It is used to compare the classification result with the actual measurement result. By using a confusion matrix, we can evaluate the classification accuracy. The confusion matrix has a column for each category of prediction and so each column is the total number of predicted data in that category. Each row is a specific category of data. To assess the efficiency of the method, specificity, recall, precision, and accuracy were used. These criteria are expressed by Equations (22)–(25), respectively.

(22) $$\mathrm{Accuracy}=\frac{TP+TN}{TP+FN+TN+FP}$$

(23) $$\mathrm{Precision}=\frac{TP}{TP+FP}$$

(24) $$\mathrm{Recall}=\frac{TP}{TP+FN}$$

(25) $$\mathrm{Specificity}=1-\frac{FP}{TN+FP}$$

As well as the actual sample category, the model recognition is positive, as indicated by *TP*. *TN* indicates that the sample category is negative, as is the model recognition. The actual sample category is negative, while the model recognition is positive, as indicated by *FP*. The actual sample category is positive, while the model identification is negative, as indicated by *FN*.

#### 4.2.2. Diagnostic Analysis

A total of 1190 sampling points were divided into a data sample. In total, 200 samples were selected from each fault category, amounting to 2000 samples. At a ratio of 4:1, the training and testing samples were divided into three multi-scale 1DCNN diagnosis subnetwork models for feature extraction, and the results were input into the improved DS evidence theory model to achieve decision-level fusion diagnosis classification.

In the experiment, 10 test results were selected as the final fault diagnosis results to enhance the experiment’s reliability. According to the developed multi-scale sensor information fusion diagnosis method, three sensor data sources were input into the parallel 1DCNN diagnosis subnetwork for preliminary feature extraction and fault classification. The network structure and parameters of the three 1DCNNs were identical. By training the 1DCNN network, the preliminary output results are obtained. The output results for the samples of each type of fault from the three sensor data sources are shown in Table 5.

After the preliminary diagnosis of the 1DCNN network, the model in this paper can diagnose all kinds of faults well, with an average diagnosis and recognition rate of more than 90%. Then, according to the fusion rules of the DS theory, the fusion analysis of three scaled data source networks was performed. Table 6 shows the results of the fusion.

By comparison, there may be conflicting data due to the different data sources, which means that the diagnostic results of the three network outputs are different. However, according to the results of the network training, the diagnosed fault type is consistent with the actual fault type, which also confirms that the 1DCNN network model has a better effect. After processing the DS theory, the fault mode representation of the network was comprehensively analyzed. Table 6 shows that diagnostic accuracy can reach 99.65–100.00%, which confirms the high accuracy and reliability of the model.

When the sensor amount increases, the diagnosis model has a higher accuracy and reliability and it is more effective.

#### 4.2.3. Analysis of Evaluation Results

**Figure 13:**
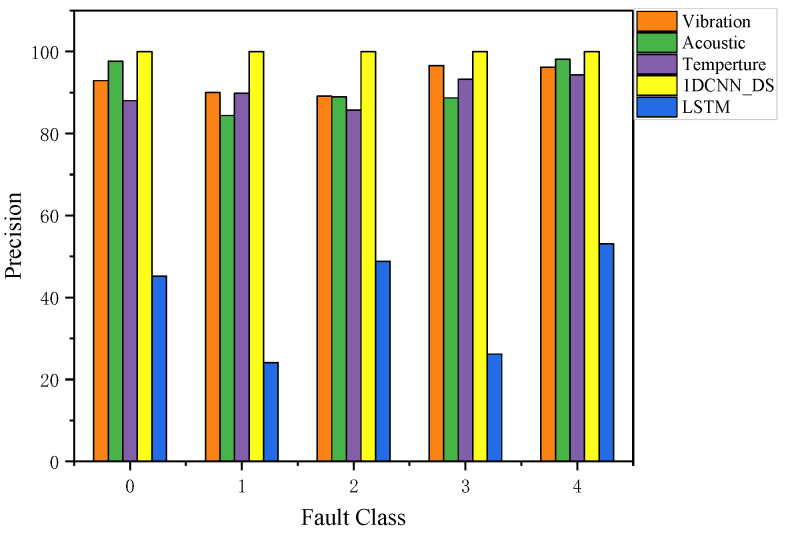
Comparison of precision.

Precision indicates how many positive class predictions are generated from positive samples: it represents the proportion of samples that are truly positive among those identified as positive by the model. Figure 13 can be used to compare the precision of all the results, including the precision of vibration, acoustic, and temperature signals after the multi-scale 1DCNN model, as well as the precision of the LSTM model and DS fusion. A model’s effect is generally better the higher its accuracy. As shown in Figure 13, a higher level of precision is achieved with 1DCNN and DS (1DCNN_DS) fusion than with the other methods. The average precision of 1DCNN_DS is 0.9934, which can meet the requirements of anomaly detection for complex industrial systems.

**Figure 14:**
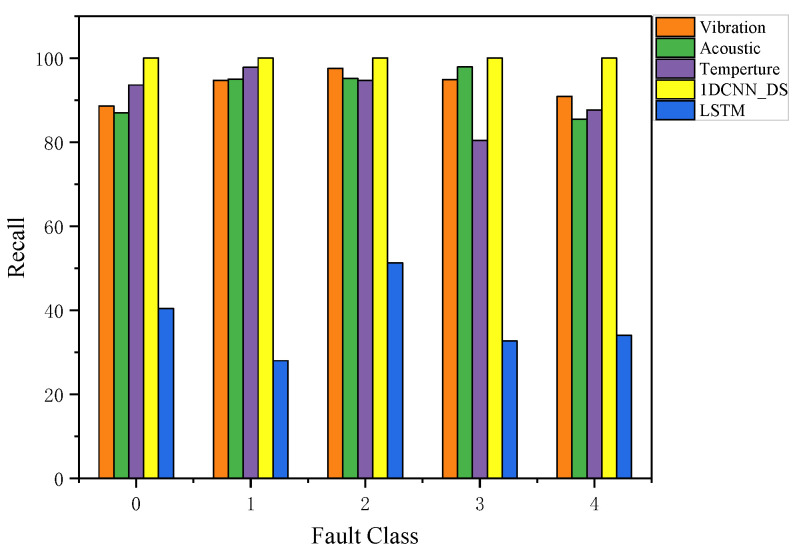
Comparison of recall.

In the dataset, recall indicates all of the positive samples which made positive predictions. It represents the ratio of positive samples identified by the model to all positive samples. Figure 14 can be used to compare the recall of all the results, including the recall of vibration, acoustic, and temperature signals after the multi-scale 1DCNN model and the recall of the LSTM model and DS fusion. A model’s effect is generally better the higher its recall. As shown in Figure 14, a higher level of recall is achieved with 1DCNN_DS fusion than with the other methods. The average recall of 1DCNN_DS is 0.9884, which is a normal and acceptable result.

**Figure 15:**
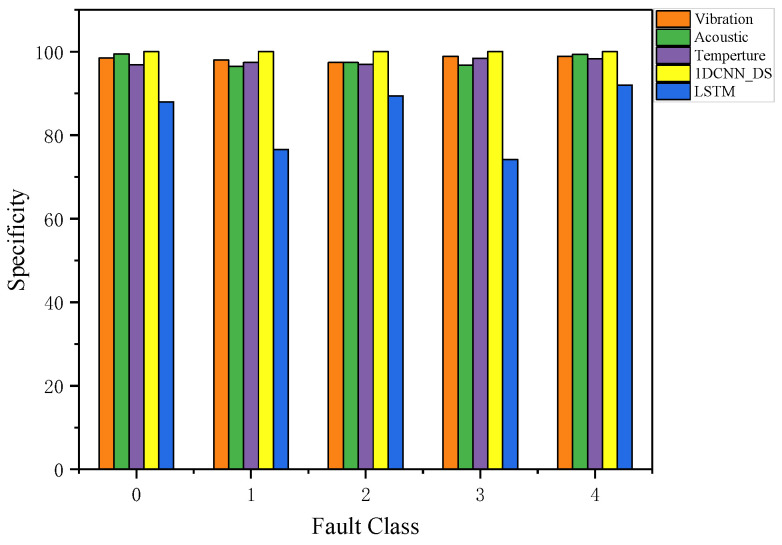
Comparison of specificity.

Specificity indicates the ratio of negative class samples the model identifies to all negative class samples. Figure 15 can be used to compare the specificity of all the results, including the specificity of vibration, acoustic, and temperature signals after the multi-scale 1DCNN model and the specificity of the LSTM model and DS fusion. A model’s effect is generally better the higher its specificity. As can be seen from Figure 15, a higher level of specificity is achieved with 1DCNN_DS fusion than with the other methods. The average specificity of 1DCNN_DS is 0.9947, which is a normal and acceptable result.

## 5. Conclusions

In this paper, a fusion network model is proposed, combining multi-scale feature extraction and the DS evidence theory in a multi-sensor scenario. It can be used to accurately and efficiently identify the fault condition in the working process of mechanical equipment. Using the one-dimensional convolutional structure to deal with one-dimensional signals in time series, the time dependence of the collected signals is maintained. On this basis, according to the training task of fault diagnosis, a CNN-based diagnostic model is developed for feature extraction of the fault signals collected by sensors. The experimental comparison shows that the developed intelligent diagnosis method 1DCNN can effectively improve the fault extraction ability.

In addition, the fusion decision fault diagnosis method with improved DS evidence theory realized the comprehensive fault analysis of devices. The diagnostic model of the DS evidence theory was built using a multi-branch 1DCNN network, and the revised body of evidence and probability distribution were established. The fault features extracted from multiple data sources were processed and fused at decision level, and the ability of the diagnostic model to handle uncertain information was improved. The proposed method improves the diagnostic accuracy, according to experimental results, which is effective and stable.

Further research can optimize the network model, build more advanced deep learning models, improve the efficiency of model training, and reduce the complexity of diagnostic models. We have developed an intelligent sensing sensor to improve the security of industrial control systems through effective configuration and the proposed fusion algorithm. In addition, effective sensor selection and fusion strategies need to be studied to improve the overall performance of the diagnostic system. This research is very meaningful in improving the safety and reliability of complex systems and reducing enterprise costs.

## Figures and Tables

**Table 1:** Parameters of input, output, and initialized pseudocode.

Parameters of Multi-Sensor Fusion Model:
**Parameter’s initialization:** Win_len: The size of each sample Step: Step size at the time of sliding window interception Batch_size(int, optional): How many samples are loaded per batch Adam_lr: Customize the learning rate $w_{i}$: Weights of convolution and pooling layer i W,b: Weights and bias of full connections layer Epochs: Number of Iterations $E_{r}$: Expected error **Input:** Dataset: Load the data set with the data, including Vibration, Acoustic, Temperature X_train, y_train: features and labels of training set X_test, y_test: features and labels of test set **1DCNN Output:** Train_loss, Test_loss: The loss value is output through the network Train_acc, Test_acc: The accuracy value is output through the network T-SNE: Dimension reduction visualization ${\tilde{y}}_{i}$: Training iteration output $M_{ij}$: Confusion matrix of each scale $J_{i}(\theta )$: The error of each iteration of training **DS Output:** $J_{j}(\theta )$: Iterative errors during training $m(A_{i})$: Diagnostic results of BPA k: Evidence conflict factor

**Table 2:** Sample data.

Fault Category	Total Samples	Training Samples	Test Samples	Label
normal	4000	3000	1000	0
bearing fault	4000	3000	1000	1
gearbox fault	4000	3000	1000	2
blade fault	4000	3000	1000	3
generator fault	4000	3000	1000	4

**Table 3:** Sample data for each type of fault.

Gearbox Fault	Bearing Fault	Generator Fault	Training Samples	Test Samples	Label
normal	normal	normal	3760	940	0
wear	wear	winding burnout	3760	940	1
shedding	fatigue	brush wear	3760	940	2
tooth breakage	shedding	bearing failure	3760	940	3
eccentric wear	sediment	mechanical component damage	3760	940	4
skew	eccentricity	unstable motor operation	3760	940	5

**Table 4:** 1DCNN model detailed parameters.

Layer	Layer Type	Kernel Size/Stride/Kernel Channel Size	Remark
1	Convolution0	32 × 1/2/16	Relu
2	Convolution1	16 × 1/2/32	Relu
3	Pooling1	2 × 1/1/32	Max pooling
4	Convolution2	8 × 1/2/64	Relu
5	Pooling2	2 × 1/1/64	Max pooling
6	Convolution3	4 × 1/2/128	Relu
7	Pooling3	2 × 1/1/128	Max pooling
8	Flattening	256	
9	Fully Connected	256	Relu
10	Softmax	5	

**Table 5:** Multi-scale 1DCNN diagnosis actual output results.

Sensor	Actual Output					Actual Category	Diagnostic Category
	0	1	2	3	4		
Vibration	0.9286	0.0000	0.0000	0.0714	0.0000	0	0
	0.0500	0.9000	0.0250	0.0000	0.0250	1	1
	0.0652	0.0000	0.8913	0.0000	0.0435	2	2
	0.0000	0.0000	0.0000	0.9655	0.0345	3	3
	0.0000	0.0385	0.0000	0.0000	0.9615	4	4
Acoustic	0.9756	0.0000	0.0000	0.0000	0.0244	0	0
	0.0222	0.8444	0.0222	0.0000	0.1112	1	1
	0.1111	0.0000	0.8889	0.0000	0.0000	2	2
	0.0000	0.0377	0.0189	0.8868	0.0566	3	3
	0.0000	0.0000	0.0185	0.0000	0.9815	4	4
Temperature	0.9574	0.0426	0.0000	0.0000	0.0000	0	0
	0.0408	0.9592	0.0000	0.0000	0.0000	1	1
	0.0333	0.0000	0.8667	0.0333	0.0667	2	2
	0.0500	0.0000	0.0000	0.9500	0.0000	3	3
	0.0000	0.0000	0.0000	0.0000	1.0000	4	4

**Table 6:** DS evidence theory fusion results.

Actual Category	$m(A_{1})$	$m(A_{2})$	$m(A_{3})$	$m(A_{4})$	$m(A_{5})$	m(Θ)	Conflict Factor (*k*)
0	1.0000	0.0000	0.0000	0.0000	0.0000	0.0000	0.1678
1	0.0001	0.9999	0.0000	0.0000	0.0000	0.0000	0.2710
2	0.0035	0.0000	0.9965	0.0000	0.0000	0.0000	0.3131
3	0.0000	0.0000	0.0000	1.0000	0.0000	0.0000	0.1866
4	0.0000	0.0000	0.0000	0.0000	1.0000	0.0000	0.0563

## Data Availability

The data used in this study are self-collected.
